# Emerging *Fusarium* and *Alternaria* Mycotoxins: Occurrence, Toxicity and Toxicokinetics

**DOI:** 10.3390/toxins9070228

**Published:** 2017-07-18

**Authors:** Sophie Fraeyman, Siska Croubels, Mathias Devreese, Gunther Antonissen

**Affiliations:** 1 Department of Pharmacology, Toxicology and Biochemistry, Faculty of Veterinary Medicine, Ghent University, Salisburylaan 133, 9820 Merelbeke, Belgium; sophie.fraeyman@ugent.be (S.F.); mathias.devreese@ugent.be (M.D.); gunther.antonissen@ugent.be (G.A.); 2 Department of Pathology, Bacteriology and Avian Diseases, Faculty of Veterinary Medicine, Ghent University, Salisburylaan 133, 9820 Merelbeke, Belgium

**Keywords:** emerging mycotoxin, occurrence, toxicity, toxicokinetics, *Fusarium*, *Alternaria*

## Abstract

Emerging *Fusarium* and *Alternaria* mycotoxins gain more and more interest due to their frequent contamination of food and feed, although in vivo toxicity and toxicokinetic data are limited. Whereas the *Fusarium* mycotoxins beauvericin, moniliformin and enniatins particularly contaminate grain and grain-based products, *Alternaria* mycotoxins are also detected in fruits, vegetables and wines. Although contamination levels are usually low (µg/kg range), higher contamination levels of enniatins and tenuazonic acid may occasionally occur. In vitro studies suggest genotoxic effects of enniatins A, A1 and B1, beauvericin, moniliformin, alternariol, alternariol monomethyl ether, altertoxins and stemphyltoxin-III. Furthermore, in vitro studies suggest immunomodulating effects of most emerging toxins and a reproductive health hazard of alternariol, beauvericin and enniatin B. More in vivo toxicity data on the individual and combined effects of these contaminants on reproductive and immune system in both humans and animals is needed to update the risk evaluation by the European Food Safety Authority. Taking into account new occurrence data for tenuazonic acid, the complete oral bioavailability, the low total body clearance in pigs and broiler chickens and the limited toxicity data, a health risk cannot be completely excluded. Besides, some less known *Alternaria* toxins, especially the genotoxic altertoxins and stemphyltoxin III, should be incorporated in risk evaluation as well.

## 1. Introduction

Mycotoxins are low-molecular-weight secondary fungal metabolites that can contaminate various food and feed commodities, including but not limited to grain and grain-based products, vegetables, fruits and fruit juices, oil seeds and oils, spices, coffee and wine [1,2]. The continuous development of liquid chromatography coupled to mass spectrometric methods makes it possible to analyze an almost non-exhaustive list of mycotoxins in a variety of food and feed matrices [3,4]. These multi-mycotoxin screenings revealed a high prevalence of so called “emerging” mycotoxins. In feed for example, the *Fusarium* mycotoxins beauvericin (BEA), enniatins (ENNs) and moniliformin (MON) were detected in 98%, 96% and 76% of the feed and feed ingredient samples (*n* = 83), respectively. Eighty-two percent, 80% and 65% of the samples analyzed between 2010 and 2012 were contaminated with the *Alternaria* mycotoxins alternariol monomethyl ether (AME), alternariol (AOH) and tenuazonic acid (TeA), respectively [4]. Although these mycotoxins are frequently detected, no legislation or guideline is available and the in vivo toxicity data is limited. In vitro studies suggest genotoxic effects of BEA, ENN A, A1, B1, MON, AOH, AME, altertoxin-II (ATX-II) and stemphyltoxin-III (STTX-III) [5,6,7,8,9,10]. Besides, MON and TeA cause severe toxicity in animals, with MON mainly affecting the heart and TeA causing hemorrhages [11,12,13,14]. Therefore, these frequently detected food and feed contaminants could pose a health risk for humans and animals. In 2014 and 2011, the European Food Safety Authority (EFSA) disclosed some knowledge gaps regarding the occurrence, toxicity and toxicokinetics of BEA, ENNs and *Alternaria* toxins. Since then, new studies focusing on occurrence, toxicokinetics and metabolism of these emerging mycotoxins have been published [15,16,17,18,19,20,21,22,23]. Therefore, the objective of this review is to give an updated overview of the occurrence in food and feed, in vitro and in vivo toxicity and toxicokinetics of BEA, ENNs, MON and the *Alternaria* mycotoxins. In addition, remaining knowledge and research gaps in this field were identified. For an overview of other emerging mycotoxins, the authors would like to refer to the work of Gruber-Dorninger et al. (2016) [24].

## 2. Beauvericin and Enniatins

**Figure 1:**
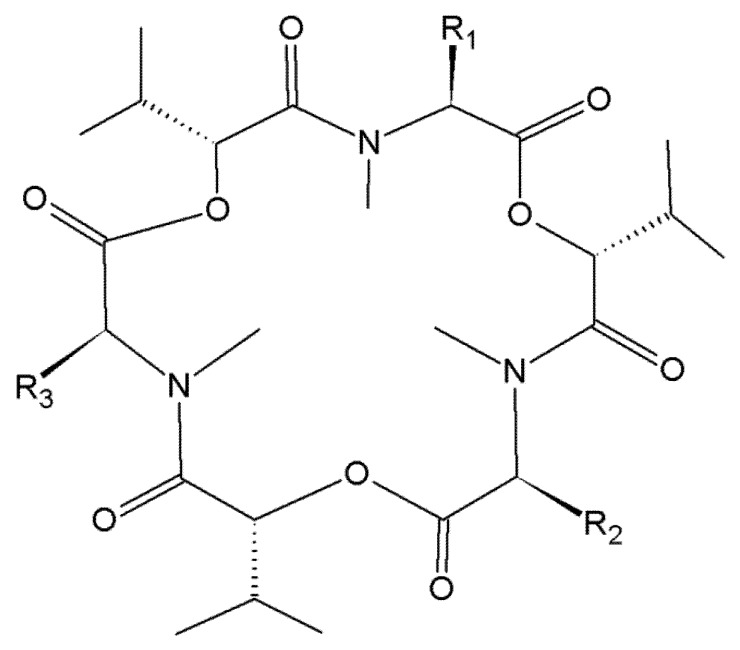
Chemical structure of beauvericin, enniatin A, A1, B and B1. Beauvericin (BEA), R1 = R2 = R3 = phenylmethyl; Enniatin A (ENN A), R1 = R2 = R3 = –CH(CH_3_)CH_2_CH_3_; Enniatin A1 (ENN A1), R1 = R2 = –CH(CH_3_)CH_2_CH_3_, R3 = –CH(CH_3_)_2_; Enniatin B (ENN B), R1 = R2 = R3 = –CH(CH_3_)_2_; Enniatin B1 (ENN B1), R1 = R2 = –CH(CH_3_)_2_, R3 = –CH(CH_3_)CH_2_CH_3._

In the search for new antibiotics, BEA was isolated for the first time from *Beauveria bassiana* as early as in 1969 [25]. BEA and ENNs are cyclic depsipeptides produced by a wide variety of *Fusarium* fungi, including but not limited to *F. acuminatum*, *F. avenaceum*, *F. oxysporum*, *F. poae*, *F. sporotrichioides*, *F. sambucinum* and *F. tricinctum* [26,27,28,29,30]. Most frequently detected mycotoxins are BEA, ENN A, A1, B and B1 (Figure 1).

### 2.1. Occurrence of Beauvericin and Enniatins in Food and Feed

BEA and ENNs can contaminate a variety of foodstuffs (Table 1). Grain-based food products are the most important contributors to the acute and chronic dietary exposure [16]. BEA contaminated the majority of Scandinavian cereals, including wheat, oats, barley and rye. Prevalence ranged between 12% and 100%. Mostly, contamination levels were below 100 µg/kg. The highest concentration detected was 220 µg/kg in barley [17,31,32]. Compared to Scandinavian cereals, Moroccan samples contained much higher BEA levels, with a maximum contamination level of 26,300 µg/kg in rice. Moroccan breakfast cereals and couscous seemed not frequently contaminated (0–2.9%), but Moroccan rice was highly contaminated (76%). However, it should be noted that the limit of quantification (LOQ) in the studies of Mahnine et al. (2011) and Sifou et al. (2011) was rather high (500 µg/kg), which at least partly could explain the lower incidence of BEA in Moroccan cereals. The differences in LOQ could be attributed to the fact that both Mahnine et al. (2011) and Sifou et al. (2011) used LC-diode array detection (DAD) to determine BEA, whereas other authors used LC-MS [17,31,32,33,34,35,36,37]. 

Regarding ENNs, almost all Scandinavian cereal samples were contaminated, with a high prevalence (range 96% and 100%). Contamination levels were usually in the µg/kg range (median concentrations 41–569 µg/kg), but could occasionally reach the lower mg/kg range. The maximum concentration was about 14,850 µg/kg in a barley sample. Scandinavian results were reported as the sum of different ENN analogues, ENN A, A1, B, B1, B2 and B3, respectively or the analogue was not specified in the presented results [17,31,32]. Similar to BEA, contamination levels for ENNs were higher in Moroccan cereals. Maximum concentrations of ENN B1 (795,000 µg/kg), ENN A1 (688,000 µg/kg), ENN A (119,500 µg/kg), and ENN B (81,100 µg/kg) were found in wheat, muesli, rice and oats, respectively [33,34]. In contrast, Moroccan couscous was not contaminated with BEA (*n* = 98, LOD = 1 µg/kg) and ENN concentrations were mostly lower than 100 µg/kg [35].

Concerning feed, the prevalence of BEA ranged between 50% and 98%, and the observed maximum concentrations were up to 2326 µg/kg. Regarding ENNs, feed contamination levels ranged between <0.1–1745, <0.15–2216, <0.3–1514 and <0.2–2690 µg/kg, for ENN A, A1, B and B1, respectively (Table 1) [4,38,39,40].

### 2.2. Toxicity of Beauvericin and Enniatins

#### 2.2.1. In Vitro Toxicity

The cytotoxic effects of BEA and ENNs have been demonstrated in a variety of cell cultures (Table 2) [5,6,52,53,54,55,56]. The mechanism of toxicity is considered to be related to their ionophoric properties. BEA and ENNs can insert into the cell membrane, forming cation-selective pores and influencing cellular ionic homeostasis. Increased intracellular Ca^2+^ can trigger cytochrome C release, which consequently induces an increased caspase-3 activity, resulting in apoptosis. Furthermore, increased intracellular Ca^2+^ can also trigger necrosis [5,6,54,57,58]. Besides, BEA and ENNs exert their cytotoxic effect through stimulating the production of reactive oxygen species (ROS) resulting in induction of lipid peroxidation (LPO) and glutathione depletion in Caco-2 cells [5,6,58]. Prosperini et al. (2013) demonstrated that ENN A and A1 were more cytotoxic compared to ENN B1 and BEA, while ENN B had the lowest cytotoxic effect in Caco-2 cells. On the other hand, IPEC-J2 cells were more sensitive to ENN B, followed by BEA, ENN B1, ENN A and ENN A1. In Caco-2 cells, ENN A, A1 and B1 induced DNA damage, resulting in a cell cycle arrest at the G2/M phase at concentrations between 1.5 µM and 3.0 µM. ENN B was not genotoxic in Caco-2 cells. BEA caused cell cycle arrest in the G2/M phase and S phase, presumably as a result of the cellular redox imbalance and at higher concentrations (12 µM), BEA caused DNA damage in Caco-2 cells. Above, 2.5–10 µM BEA promoted chromosome aberrations, increased the frequency of sister-chromatid exchanges and induced micronuclei in human lymphocytes (Table 2) [5,6,7,59]. 

The neurotoxic and myotoxic effects of BEA were demonstrated in a mouse hemidiaphragm preparation. At low concentrations (5 µM), BEA depressed acetylcholine release presynaptically, but at higher concentrations (7.5 and 10 µM), BEA had a direct effect on skeletal muscle fibers, resulting in contractures [60]. 

Additionally, limited in vitro studies also suggest a toxic effect of BEA and ENNs on the reproductive system (Table 2). BEA impaired the development of cultured porcine oocytes and early embryos. Exposure to BEA resulted in a decreased progesterone synthesis in cumulus cells, reduced MDR1 activity as a consequence of ATP depletion in zygotes and decreased mitochondrial activity in early embryos [61]. Besides, BEA inhibited estradiol and progesterone synthesis in bovine granulosa cells by suppressing *CYP19A1* and *CYP11A1* gene expression [62]. ENN B also reduced progesterone, testosterone and cortisol secretion in human adrenocortical carcinoma cells (H295R) and modulated the expression of genes involved in steroidogenesis [63].

Furthermore, BEA and ENNs exert immunomodulating effects (Table 2). Ficheux et al. (2013) demonstrated that BEA and ENN B increase IL-10 secretion and affect the initiation of the adaptive immune response by interfering with dendritic cell migration. Moreover, endocytosis by macrophages was decreased after exposure to BEA and ENN B [53]. Besides, ENNs and BEA could also affect health by antimicrobial effects on both pathogenic and probiotic microorganisms. BEA inhibited the growth of pathogenic bacteria, such as *Escherichia coli*, *Enterococcus faecium*, *Salmonella enterica*, *Shigella dysenteriae*, *Listeria monocytogenes*, *Yersinia enterocolitica*, *Clostridium perfringens* and *Pseudomonas aeruginosa* [64]. On the other hand, ENN A, A1 and B1 could also inhibit the growth of probiotic microorganisms. Roig et al. (2014) demonstrated the growth inhibition of *Streptococcus thermophilus* and different strains of the genus *Bifidobacterium* and *Lactobacillus* by ENN A1 and ENN B1, while ENN A inhibited *Saccharomyces cerevisiae*. No impact of ENN B was observed on the growth of different probiotic microorganisms [65]. Further studies on the antimicrobial effects of BEA and ENNs are limited, however, indicating a potential impact on the intestinal microbiota. 

#### 2.2.2. In Vivo Toxicity

The in vitro observed immunomodulating effects of BEA and ENNs have been confirmed in rodents. ENN A modulated surface antigen expression of peripheral blood lymphocytes. The number of T-helper (CD4^+^) cells increased, while cytotoxic T-cells (CD8^+^) decreased in Wistar rats fed an ENN A contaminated feed (465 mg/kg) for 28 days [76]. BEA decreased serum levels of tumor necrosis factor (TNF-)α and interferon (IFN-)γ, and induced apoptosis of activated T-cells of mice with experimental colitis after intraperitoneal (ip) administration (2–4 mg/kg) [77]. Fusafungine, a nose and mouth spray consisting of a mixture of ENNs, was used to treat upper airway infections. Mice exposed to fusafungine oral spray, showed low-grade dysplasia, congestion and edema on the tongue, hyperplasia of the cheek mucosa and low-grade dysplasia of the superficial epithelium [78,79]. Recently, the European Medicines Agency (EMA) has revoked the authorization of fusafungine sprays, since the benefits do not outweigh the risks, especially the risk of serious allergic reactions [79]. On the other hand, a daily intake of 20.91 mg ENN A/kg bw/day during 28 days did not cause any adverse effects in Wistar rat. The ENN A contaminated diet was obtained through infection with *F. tricinctum* [80]. However, the EFSA CONTAM Panel concluded that the control and contaminated feed were not comparable due to fungal growth and that there were insufficient details on the detection methods used and on the occurrence of other mycotoxins in the feed to draw conclusions [16].

In vivo toxicity data of BEA and ENNs in livestock and companion animals is limited to poultry. Broiler chickens and laying hens were fed a multi-mycotoxin contaminated diet containing DON (1710–2228 µg/kg), HT-2 (488–606 µg/kg), T2-toxin (367–343 µg/kg), ZEN (753–820 µg/kg), 3-ADON (41–66 µg/kg), 15-ADON (91–227 µg/kg), ENN A (28 µg/kg), ENN A1 (491–440 µg/kg), ENN B (12,716–11,233 µg/kg), ENN B1 (4057–3599 µg/kg) and BEA (10,313–8926 µg/kg) for 14 days. No impact on animal performances, such as growth, feed uptake and egg production was observed [81]. 

Feeding a multi-mycotoxin contaminated diet containing up to 2.7 mg/kg MON and up to 12 mg/kg BEA showed no significant effects on growth or carcass traits of broiler chickens. No residues were detected in the carcass and organs. However, no details on the analytical methods used to determine the concentration of the mycotoxins in the feed, carcass and organs were described. Since no LOQ was reported, no conclusions could be drawn regarding tissue residues. Likewise, growth and slaughter performance were not affected in broilers and turkeys fed a multi-mycotoxin contaminated diet containing both MON and BEA. Contamination levels were as high as 0.5 mg/kg MON and 0.8 mg/kg BEA in broiler feed, and 3.0 mg/kg MON and 2.48 mg/kg BEA in turkey feed [16,82].

The EFSA Panel on Contaminants in the Food Chain (CONTAM) identified no-observed-adverse-effect levels (NOAELs) for BEA for broiler chickens (1220 µg/kg bw/day), laying hens (536 µg/kg bw/day) and turkeys (136 µg/kg bw/day). The identified NOAELs for ENN B and B1 were 763 and 244 µg/kg bw/day for broiler chickens and 674 and 216 µg/kg bw/day for laying hens [16]. However, a proper risk assessment of chronic toxicity in livestock other than poultry and in companion animals is impossible due to the lack of LOAELs and NOAELs. Furthermore, the EFSA CONTAM panel concluded that the lack of toxicity data precludes a risk assessment for dietary exposure of humans to BEA and ENNs [16].

### 2.3. Toxicokinetics of Beauvericin and Enniatins

Besides exposure and toxicity data, knowledge of toxicokinetics is indispensable for a proper risk assessment, since oral bioavailability, rate of absorption, (pre-)systemic biotransformation, distribution and excretion influence the internal dose of a compound [83]. Toxicokinetic analysis demonstrated the high absolute oral bioavailability (F = 90.9%) of ENN B1 in pigs [20]. In contrast, ENN B1 and ENN B were poorly absorbed after oral administration to broiler chickens, with an F of 5% and 11% for ENN B1 and ENN B, respectively [21]. Both total body clearance (Cl) and volume of distribution (V_d_) of ENN B1 were higher in broiler chickens compared to pigs. The Cl of ENN B1 after iv administration was 6.6 L/h/kg and 1.91 L/h/kg in broiler chickens and pigs, respectively. The V_d_ of ENN B1 was 25 L/kg in broiler chickens. In pigs, the toxicokinetics fitted a two-compartmental model with a V_d_ of the central and peripheral compartment of 0.57 and 0.69 L/kg, respectively. The low oral bioavailability and rather high clearance of ENN B and B1 in chickens is in accordance with the EFSA statement that chronic and acute adverse health effects associated with BEA and ENNs in poultry are unlikely [16,21]. 

Remarkable species differences in oral bioavailability and toxicokinetic parameters could be attributed to differences in biotransformation. Hydroxylated and carboxylated metabolites of ENN B and B1 have been identified in plasma of poultry and pigs. Additionally, carbonylated metabolites of ENN B1 have been detected in pig plasma. Metabolite/ENN B1 ratios were higher after oral, compared to intravenous administration, indicating presystemic metabolism of ENN B1 in pigs. Presystemic metabolism could not be distinguished from systemic metabolism in broiler chickens, due to the low parent and metabolite plasma levels after oral administration [21,23,84]. Besides presystemic metabolism, elimination can also occur through efflux into the gut lumen via ATP-binding cassette (ABC) transporters. The major ABC transporters are P-glycoprotein (P-gp, multidrug resistance protein 1, MDR 1), multidrug resistance-associated protein 2 (MRP 2) and breast cancer resistance protein (BCRP) [85,86]. Induction or inhibition of ABC transporters may not only affect oral bioavailability and absorption, but the entire kinetic profile of toxins and drugs, mainly through their effect on hepatic and renal Cl and V_d_ since they are located in these organs as well [87,88]. In vitro studies using human cell lines suggest that ABC transporters may play an important role in the absorption of ENNs and BEA, and consequently influencing their oral bioavailability. Ivanova et al. (2010) suggested that P-gp, MRP 2 and BCRP could be involved in the efflux of ENN B1 into the intestinal lumen. Besides, overexpression of BCRP and P-gp resulted in a significant resistance towards the cytotoxic effects of ENNs and BEA in vitro. Above, chronic exposure to stepwise increasing ENN or BEA concentrations resulted in an upregulation of ABC-transporter proteins and cross-resistance to other chemotherapeutics in human cell lines [89]. Interestingly, monensin, an ionophoric coccidiostat which is frequently mixed into poultry feed, can upregulate P-gp expression in the duodenum of broiler chickens [85,90]. Therefore, it could be investigated whether feed supplementation with monensin influences the absorption of ENNs and BEA by upregulating the ABC efflux transporters. In addition, ENNs and BEA reduced the intestinal barrier integrity in vitro. Reduced intestinal barrier integrity could not only affect bioavailability of xenobiotics, but could also increase the susceptibility to diseases and reduce performance [59,89]. Taken together, the toxicokinetic behavior of these mycotoxins may be complex and can vary between both individuals and species, making species- and compound-specific studies essential.

Data regarding the carry-over of BEA and ENNs in food of animal origin is limited. Finnish eggs were frequently contaminated with BEA and/or ENNs (56–99.7%). Although levels were low for most of the egg samples, co-contamination of at least two toxins occurred regularly in the yolk samples (77%). Both whole egg and yolk samples were most frequently contaminated with ENN B (93–46% of positive samples) and BEA (7–31% of positive samples), but ENN A and ENN A1 were only detected in 1% and 2% of the positive yolk samples and were not detected in whole egg samples. The higher contamination in yolk, compared to whole eggs, could be attributed to the lipophilic nature of ENNs and BEA [91]. In contrast with the Finnish survey (*n* = 479 eggs), neither BEA nor ENNs were detected in commercial eggs (*n* = 30) or in pig meat (*n* = 10) obtained from the Belgian market. In chicken meat samples, only traces of BEA and/or ENNs were detected and ENN B was detected in 4/16 pig liver samples. It should be noted that the number of samples in the Belgian survey was limited and no LOQ was reported for the determination of BEA and ENNs in the different Belgian samples. [16,81]. Callebaut et al. (2011/2012) studied the carry-over of mycotoxins to poultry products by administrating a multi-mycotoxin contaminated diet to both laying hens and broiler chickens. In broiler chickens, traces of ENN A and ENN A1 were only detected in meat, however ENN B, ENN B1 and BEA were detected in meat, liver and skin. Carry-over rates for ENN B and B1 were the highest in skin (0.39% and 0.37%, respectively), followed by liver (0.16% and 0.12%, respectively), thigh muscle (0.04%) and breast muscle (0.01% and 0.025%, respectively). Carry-over rate for BEA was highest in liver (1.57%), followed by skin (1.16%) and muscle (0.03%). ENNs and BEA were rapidly eliminated from liver and meat, but elimination was slower and incomplete in skin tissue. Regarding eggs, ENN B, B1 and BEA were detected at 2 to 3 days after the contaminated feed was first administrated and reached a maximum at day 5. It took up to 10 withdrawal days, during which the hens received a control diet, to eliminate ENNs and BEA from the eggs. Carry-over rates were highest for BEA (0.44%), followed by ENN B (0.10%) and ENN B1 (0.05%) [81]. BEA was not detected in milk, sausage, pork or pig liver [38].

## 3. Moniliformin

**Figure 2:**
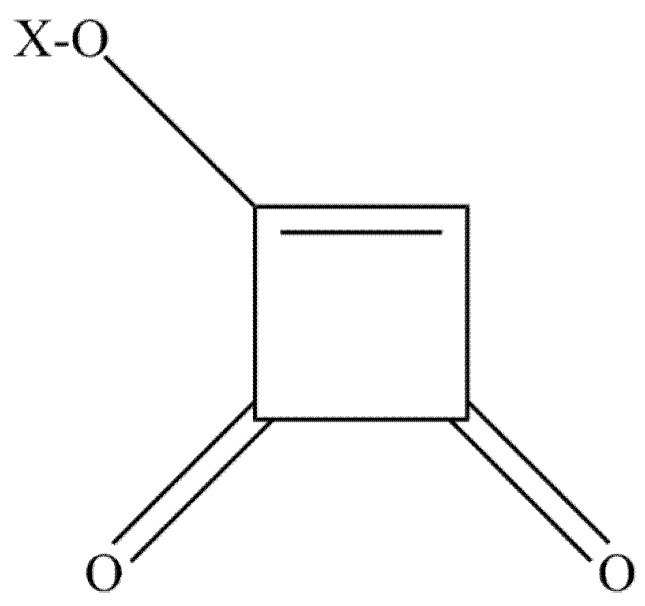
The chemical structure of moniliformin. X=H, Na or K.

Moniliformin (MON) or 1-hydroxycyclobut-1-ene-3,4 dion (Figure 2) was first identified as a mycotoxin of *F*. *moniliforme*, now referred to as *F. verticillioides* [92,93,94]. Besides, MON can also be produced by *F*. *begoniae*, *F*. *denticulatum*, *F*. *lactis*, *F*. n*i*sikadoi, *F*. *phyllophilum*, *F*. *pseudocircinatum*, *F*. *pseudonygamai*, *F*. *ramigenum*, *F*. *tricinctum*, *F*. *acutatum*, *F*. *anthophilum*, *F*. *bulbicola*, *F*. *concentricum*, *F*. *diaminii*, *F*. *fujikuroi*, *F*. *napiforme*, *F*. *nygamai*, *F*. *proliferatum*, *F*. *pseudoanthophilum*, *F*. *sacchari*, *F*. *subglutinans*, *F*. *thapsinum*, *F*. *beomiforme*, *F*. *oxysporum*, *F*. *redolens*, *F*. *chlamydosporum*, *F*. *arthrosporiodes*, *F*. *avenaceum* and *F*. *acuminatum*. Above, *Penicillium melanoconidium* can also produce MON [30,95,96,97].

### 3.1. Occurrence of Moniliformin

Data on the occurrence of MON in food is limited and summarized in Table 1. In European grain samples, contamination levels ranged between <15–2606 µg/kg and the prevalence ranged between 0.88–100%. MON levels were usually below 100 µg/kg. The maximum MON concentration of 2606 µg/kg was detected in Italian maize. However, a relatively high concentration of MON (2078 µg/kg) was also detected in a Swedish wheat [17,31,32,41]. 

The prevalence of MON in feed was high, namely 76–79%, as reported by Kovalsky et al. and Streit et al. (2013). In contrast, only 3% of the Brazilian poultry feed samples was contaminated with MON. Reported maximum concentration from worldwide feed samples was 12,236 µg/kg. [4,39,40]. 

### 3.2. Toxicity of Moniliformin

The in vitro and in vivo toxic effects of MON are summarized in Table 2 and Table 3, respectively.

MON exerts its toxic effects by inactivating thiamine enzymes, including pyruvate dehydrogenase. Pyruvate dehydrogenase contributes to the formation of acetyl-CoA, which is used in the Krebs cycle. Consequently, MON compromises cellular energy supply [105].

In vitro half maximal inhibitory concentration (IC_50_) of MON ranged between 24 and >100 µg/mL in different cell lines [66]. MON negatively affect immune system by disturbing monocyte differentiation into dendritic cells and macrophages [53]. Furthermore, MON was clastogenic in human lymphocytes and caused chromosomal aberrations, an increase in sister chromatid exchanges and an increase in micronuclei frequency in a dose-dependent manner [8] (Table 2).

MON is strikingly toxic in vivo (Table 3). The heart is the main target organ of MON, causing acute heart failure, but the mycotoxin can also cause muscle weakness, respiratory distress and negatively affect immunity and animal performance [11,12,98,103,104]. MON is acutely toxic and has a narrow range of toxicity with an LD_50_ cut-off value of 25 mg/kg bw in rats. Similarly, 56% of the broiler chickens fed a diet contaminated with 200 mg MON/kg feed died. Assuming that broilers have a daily feed consumption of 100 g feed/kg bw, the LD_50_ in poultry and rats is similar [11,98]. A 28-day subacute toxicity study demonstrated that low oral doses of MON (3–6 mg/kg bw) did not cause clinical symptoms in rats. Higher doses caused somnolence and muscle weakness. Two animals in the highest dose group died of acute heart failure. The phagocytic activity of neutrophils was decreased in all treatment groups (3–15 mg/kg bw) even up to 14 days after the last administration, suggesting a prolonged inhibiting effect of MON on the innate immune system [99]. In poultry, chronic dietary exposure to MON negatively influenced the immune response and performance. Cardiotoxicity and hepatotoxicity of MON have been observed in turkeys at contamination levels of 25 mg/kg and 37.5 mg/kg feed, respectively. Higher mortality rates, lower feed conversion rates, higher heart and proventriculus weights and heart lesions were observed in broilers fed a MON contaminated diet of 50 mg MON/kg feed [102,103]. Similarly, Japanese quails fed a diet containing MON suffered from cardiac lesions (Table 3). When the feed was co-contaminated with fumonisin B1, more severe lesions were observed. Interestingly, a massive increased number of mitochondria was associated with the disruption of heart muscle fibers. This increase of the number of mitochondria could be a compensatory event for the decreased cellular energy, since MON compromises the Krebs cycle [12,105]. It should be noted that the experimental MON levels in the feed studies were about a factor 1000 higher (50–200 mg/kg) than the levels usually found in feed samples (µg/kg range). However, high contamination levels (up to 12 mg/kg), approaching the hepato- and cardiotoxic levels for poultry (25–50 mg/kg), occasionally occur [4,102].

### 3.3. Toxicokinetics of Moniliformin

The toxicokinetic behavior of MON is largely unknown. MON did not affect intestinal nor blood capillary integrity in vitro. However, in vitro studies suggest that the molecule can pass the blood brain barrier [59,106]. After single oral gavage, 42% of the administered MON was excreted in the urine of rats within 24 h post administration, and less than 1% was excreted in the feces. No other toxicokinetic data were reported in this study [98]. To the best of the authors’ knowledge, no residue studies of MON in animal-derived products have been described.

## 4. *Alternaria* Mycotoxins

**Figure 3:**
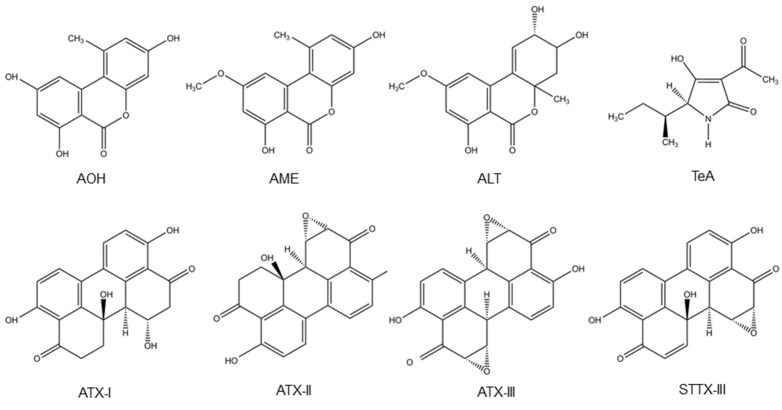
Structure of the *Alternaria* mycotoxins alternariol (AOH), alternariol monomethyl ether (AME), altenuene (ALT), tenuazonic acid (TeA), altertoxins- (ATX-) I, II and III and stemphyltoxin III (STTX-III).

*Alternaria* fungi contaminate a wide variety of food and feed crops and produce several toxins, with AOH, AME, TeA, altenuene (ALT) and altertoxins (ATXs) being the most important ones [2]. *A*. *alternata* and *A*. *arborescens* species produce TeA, ALT, AOH and AME. Besides, *A*. *alternata* also produces stemphyltoxin III (STTX-III) [107,108]. Furthermore, TeA is also produced by *A. bertholletius*, *A. caelatus*, *A. nominus*, *A*. *pseudonominus*, *A*. *arachidicola* and *A*. *bombycis* [109]. Their chemical structure is presented in Figure 3.

### 4.1. Occurrence of Alternaria Mycotoxins

Contamination of food and feed samples with *Alternaria* mycotoxins is ubiquitous in grains, fruits, vegetables and wines, as summarized in Table 1. Especially TeA is omnipresent in dried figs, sunflower seeds and tomato products [18,19,36,42,46,47,48]. Grain samples (wheat, maize and cereals) were most frequently contaminated with TeA (15–100%), followed by TEN (77%), AOH (2.4–31%), AME (3–26%), ALT (2.6–7%) and ATX-I (2.4%). For most *Alternaria* mycotoxins, contamination levels in grains were <100 µg/kg and maximum concentrations were <1000 µg/kg. However, the maximum observed TeA contamination level in wheat was 4224 µg/kg [36,42,43,44,45]. The majority of sunflower seeds, collected in retail stores in the Netherlands in 2013–2014, were contaminated with TeA with maximum concentrations of 1400 µg/kg. TEN contaminated 20–91% (maximum concentration of 0.8 mg/kg), while AOH and AME were found in 10–64% of the sunflower seeds at low concentrations (<50 µg/kg). It should be noted that the total number of samples was limited to 15 [46,47]. Although no *Alternaria* toxins were found in fresh tomatoes, most tomato products were contaminated with at least one *Alternaria* mycotoxin. TeA was detected in all tomato concentrates and almost all tomato sauces (78–100%), pastes (80%) and juices (50–100%). Furthermore, about half of the tomato pieces (60%) and ketchup (40%) samples were positive for TeA. The maximum TeA levels in tomato products ranged between 100 and 462 µg/kg. AOH (28–86%), AME (20–78%) and TEN (21–64%) were also frequently detected in tomato products, but contamination levels were usually as low as a few µg/kg. Plants can metabolise mycotoxins, forming toxins conjugated with sulphates or sugar moieties. The conjugated metabolites, AOH-3-sulfate and AME-3-sulfate, were found in tomato juices, sauces and concentrates in concentrations up to 10 µg/kg. Glucosides of AOH and AME were not detected in figs, sunflower or tomato products [18,19,46,47]. Besides, most *Alternaria* toxins were also detected in fruit juices and wines. Contamination levels were usually a few µg/L. TeA was found in the highest concentrations, namely 250 µg/L in juices, 60 µg/L in white and 46 µg/L in red wine. In contrast, ATX-I and ATX-II were not detected in bakery products, wines, juices or vegetable oils. However, the number of samples was limited, which could be a bias explaining the absence of less frequently occurring toxins [18,19,48]. Noteworthy, all infant food products were contaminated with TeA with levels ranging between 0.8–1,200 µg/kg. Highest contamination levels were found in sorghum-based infant cereals (mean 550 µg/kg) [49].

Similar to food samples, the prevalence of *Alternaria* mycotoxins in feed samples varies enormously with contamination levels between 0–80% for AOH and 1.5–82% for AME. Maximum levels were 221 µg/kg and 733 µg/kg for AOH and AME, respectively. Sixty-five percent of feed samples were contaminated with TeA up to 1983 µg/kg [4,50,51].

### 4.2. Toxicity of Alternaria Mycotoxins

The toxicity data of *Alternaria* toxins are summarized in Table 2 and Table 3. AOH and AME are cytotoxic and induce apoptotic cell death through the mitochondrial pathway [67,68,69]. Recently, the mechanisms of AOH toxicity were reviewed by Solhaug et al. (2016) [110]. AOH forms ROS and interacts with DNA topoisomerase, causing single and double DNA strand breaks. Cell cycle arrest in G2/M-phase, possibly in an attempt to repair the DNA damage, causes a decrease in proliferation. Similarly, AME is also mutagenic and causes DNA strand breaks and cell cycle arrest [9,10,55,67,68,110,111]. The quinones ATX-II and STTX-III are much stronger mutagens compared to AOH [9,10]. Under cell free conditions, ATX-II, STTX-III and AOH inhibit topoisomerase IIα. While AOH acts as a topoisomerase poison, ATX-II and STTX-III are catalytic inhibitors. AOH, but not ATX-II and STTX-III, causes double DNA strand breaks. It is suggested that ATX-II and STTX-III induce DNA damage through the formation of adducts via epoxide groups [10]. 

Moreover, AOH acts immunomodulating in THP-1 monocytes by interfering with macrophage differentiation and decreasing TNF-α secretion. AOH induced morphological changes and modified the phenotype in both RAW 264.7 mouse and primary human macrophages. Endocytic activity and autophagy were increased in RAW 264.7, but decreased in primary human macrophages. Furthermore, RAW 264.7 macrophages entered senescence following prolonged exposure to AOH (48–72 h, 30–60 µM) [55,75,110,112].

The dibenzo-α-pyrones AOH, AME and ALT structurally resemble estradiol. AOH exhibits an estrogenic response and interferes with steroidogenesis [70,72,73]. Progesterone and estradiol levels and progesterone receptor expression were increased by the estrogenic action of AOH in human adrenocortical carcinoma cells and transformed human mammary gland cells [72]. In contrast, progesterone secretion and cell viability were negatively affected by both AOH and AME, but not TeA in porcine granulosa cells. Furthermore, AOH and AME decreased the abundance of the key enzyme in progesterone synthesis, i.e. P450 cholesterol side-chain cleavage enzyme (P450SCC), but not the corresponding gene transcript (*Cyp11a1*) [71]. In vivo studies on the effects of *Alternaria* mycotoxins on reproductive and developmental health are limited. AME (200 mg/kg bw, ip) was maternally toxic and fetotoxic to Syrian golden hamsters, but did not cause teratogenic malformations [100]. In a chicken embryo assay, AOH, AME and ALT did not cause mortality, difference in weight of hatched chicks or teratogenic effects at doses up to 1000; 500 and 1000 µg/egg, respectively [101]. 

TeA exerts its toxic effect through the inhibition of the release of newly formed proteins from the ribosomes [113]. Although in vitro studies are limited, they suggest a low in vitro toxicity of TeA [114]. However, its in vivo effects are more severe. TeA caused emesis, salivation, tachycardia, hemorrhages and hemorrhagic gastro-enteropathy in rats, mice, dogs and monkeys. Similarly, hemorrhages were also observed when feeding broiler chickens and laying hens a diet contaminated with high TeA levels. Oral LD_50_ of tenuazonic sodium salt ranged between 81–186 mg/kg bw in mice and rats. In a chicken embryo assay, the LD_50_ for TeA was 548 µg/egg, but TeA did not cause teratogenic effects at doses ranging between 150 and 1500 µg/egg [13,14,101]. 

In 2011, EFSA published a scientific opinion on the risks for animal and public health related to the presence of *Alternaria* toxins in feed and food. A threshold of toxicological concern (TTC) of 2.5 ng/kg bw/day was established for AOH and AME. Since there is no evidence of genotoxic action, the TTC for TeA was set at 1500 ng/kg bw/day. The mean and 95th percentile dietary exposure exceeded the TTC for AOH (factor 16 and 33) and AME (factor 2 and 6), but not for TeA (factor 0.009) [15,18]. Recently, the exceeding of the TTC for AOH and AME, through the consumption of bakery products, juices and tomato products has been confirmed for the German and Belgian population [18,19]. However, this EFSA opinion could be considered out of date, since more occurrence data are now available. Especially for TeA, the number of left censored data is decreased and the prevalence of this mycotoxin is high in a variety of food products like wine, figs, grain and tomato products (Table 1). In accordance with the EFSA opinion, both the mean and the 95th percentile dietary exposures via the consumption of tomato products were recently found to be below the TTC for TeA with a factor of 0.07 and 0.5, respectively [19]. However, TeA not only contaminates tomato products. A more comprehensive study including bakery products, juices, tomato products and sunflower seeds resulted in an 95th percentile dietary exposure exceeding the TTC for TeA, AOH and AME, with a factor 1.4, 12 and 60 respectively [18]. However, it needs to be stressed that for infants the dietary exposure to TeA is estimated to exceed the TTC with a factor of 2.4 due to the high contamination of sorghum/millet based cereals [115].

### 4.3 Toxicokinetics of Alternaria Mycotoxins 

Fraeyman et al. (2015) demonstrated the complete oral bioavailability of TeA in both pigs and broiler chickens. Furthermore, the total body Cl of TeA in pigs (0.45 L/h/kg) and broiler chickens (0.06 L/h/kg) was rather low. The low V_d_ in both broiler chickens (0.2 L/kg) and pigs (0.3 L/kg) could indicate a limited tissue distribution. However, especially in broiler chickens, the low Cl could possibly result in accumulation of TeA when animals are regularly fed a contaminated diet, possibly compromising animal health and elevating the risk for carry-over into the food chain. Therefore, tissue residue studies are recommended [22]. Likewise, the oral bioavailability of TeA is probably also high in humans, since almost 90% of the ingested dose was recovered in the urine of two volunteers [116]. In contrast, a recent kinetic study in mice suggests a low systemic absorption of AOH, since 90% of the total dose was excreted via the feces and up to 9% via the urine [117]. 

To the best of the authors’ knowledge, no data is available regarding carry-over of *Alternaria* mycotoxins into animal derived products. However, Asam et al. (2013) demonstrated the exposure of humans to TeA through the consumption of animal-derived products, since human urine tested positive for TeA, when the diet was restricted to cheese, milk and milk products [116].

## 5. Research Gaps

Taken together, contamination levels of these emerging *Fusarium* and *Alternaria* mycotoxins are usually low (µg/kg range). However, higher contamination levels of enniatins and tenuazonic acid may occasionally occur. In vitro studies suggest genotoxic effects of enniatins A, A1 and B1, beauvericin, moniliformin, alternariol, alternariol monomethyl ether, altertoxins and stemphyltoxin-III. Furthermore, in vitro studies suggest immunomodulating effects of most emerging toxins and a reproductive health hazard of alternariol, beauvericin and enniatin B.

As can be concluded from this literature review, there are still remaining knowledge gaps regarding the studied emerging *Fusarium* and *Alternaria* toxins in all three key factors for a proper risk assessment, including occurrence, toxicity and toxicokinetic data. Concerning the occurrence data, especially data on emerging toxins in animal-derived products and conjugated *Alternaria* mycotoxins in different food samples is lacking. An evaluation of the carcinogenic risk of the emerging *Fusarium* and *Alternaria* mycotoxins for humans by IARC is advisable. Furthermore, effects on reproductive health and immune system demonstrated in in vitro studies should be verified in vivo. Special attention should be paid to the combinatory effects of emerging mycotoxins and other immunomodulating (e.g., DON or pathogens) and estrogenic (e.g., ZEN or phytoestrogens) substances. Besides, toxicokinetic studies on MON are lacking and toxicokinetics of ENNs, BEA, AOH, AME and TeA are too limited to estimate tissue residues. Therefore, there is a gap between the in vitro toxicity data and the in vivo effect of these mycotoxins. Taking into account new occurrence data for TeA, especially in infant food, the complete oral bioavailability and low total body Cl in animals and the limited toxicity data, a health risk cannot be completely excluded. Above, several studies indicate a dietary exposure above the TTC of 1500 ng/kg bw/day [15,18,115]. Therefore, a new risk evaluation, including vulnerable populations by EFSA may be considered. Besides, the cardiotoxic MON and the possible genotoxic compounds ATXs and STTX-III may be added to the risk evaluation. 

## Figures and Tables

**Table 1:** Occurrence of emerging *Fusarium* and *Alternaria* mycotoxins in food and feed.

Commodity	Mycotoxin	Total # Samples Analyzed	Prevalence Range (%)	Concentration Range (µg/kg)	Origin of Sample with Maximum Concentration	Reference
Cereals (unprocessed) ^a^	BEA	482	12–100	<10–327	Sweden	[17,31,32]
	ENNs ^b^	482	96–100		Finland	[17,31,32]
	MON	590	0.88–100	<15–2606	Italy	[17,31,32,41]
	AOH	1582	2.4–47	0.75–832	Germany	[42,43,44,45,46]
	AME	1582	3.1–7.1	0.3–905	Germany	[42,43,44,45,46]
	TeA	1526	15–68	0.1–4224	Germany	[42,43,44]
	ALT	1106	2.63–7.1	6–196.6	Germany	[42,45]
	ATX-I	42	2.4	43	South Africa	[45]
	TEN	370	77	0.4–258.6	China	[44]
Rice	BEA	70	75.7	3800–26,300	Morocco	[34]
	ENN A	70	22.8	8400–119,500	Morocco	[34]
	ENN A1	70	5.7	56,200–448,700	Morocco	[34]
	ENN B	70	30	4400–26,200	Morocco	[34]
	ENN B1	70	24.3	3600–23,700	Morocco	[34]
Cereal products	BEA	354	0–17.5	0.1–10,600	Morocco	[33,35,36,37]
	ENN A	354	2.9–77	0.5–29,700	Morocco	[33,35,36,37]
	ENN A1	354	30–100	0.25–688,000	Morocco	[33,35,36,37]
	ENN B	354	13.2–100	0.5–81,100	Morocco	[33,35,36,37]
	ENN B1	354	17.6–100	0.5–795,000	Morocco	[33,35,36,37]
	AOH	83	31–89	8–121	Italy	[18,36]
	AME	83	26–89	<0.4–48	Italy	[18,36]
	TeA	9	100	<100–210	Germany	[18]
	TEN	9	100	<1.6–12	Germany	[18]
Tomato products ^c^	AOH	187	28–70.6	<2–41.6	Belgium	[18,19,46,47]
	AME	187	20–79	<0.9–7.8	The Netherlands	[18,19,46,47]
	TeA	187	40–100	<5–462	The Netherlands	[18,19,46,47]
	ALT	83	32–56	6.1–62.0	Belgium	[19]
	TEN	117	21–64	<3.9–8.9	Belgium	[18,19]
	AOH-3-sulfate	83	11–26	2.6–8.7	Belgium	[19]
	AME-3-sulfate	83	32–78	1.7–9.9	Belgium	[19]
Fruit juices ^d^	AOH	101	15–100	<0.2–16	Germany	[18,48]
	AME	101	25–100	<0.13–4.9	Germany	[18,48]
	TeA	101	8–100	<1.1–250	Germany	[18,48]
	ALT	101	4.3–100	1.18–18.4	Germany	[18,48]
	TEN	101	22–100	<0.5–10.27	Germany	[18,48]
	altenuic acid	78	100	2.71	Germany	[48]
	altenuisol	78	8–50	<0.74–5.58	Germany	[48]
Infant food ^e^	TeA	40	100	0.8–1200	Germany	[49]
Wines	AOH	30	20–93	0.65–11	The Netherland	[46,48]
	AME	25	93	0.8–1.45	Germany	[48]
	TeA	25	60–100	<1–60	Germany	[48]
	TEN	25	71	1.01–1.47	Germany	[48]
	altenuic acid	25	21–64	<1–6.1	Germany	[48]
	altenuisol	25	55–71	<0.74–2.91	Germany	[48]
Dried figs and olives	AOH	14	7	8.7	The Netherlands	[47]
	TeA	19	10–100	5.3–2345	The Netherlands	[46,47]
Vegetable oil	AOH	19	47	≤6	Germany	[18]
	AME	19	84	<1.1–14	Germany	[18]
	TeA	19	21	15	Germany	[18]
	TEN	19	47	<6.6–11	Germany	[18]
Sunflower seeds and oils	AOH	35	10–55	<4.9–39	Germany	[18,47]
	AME	35	9–64	<0.5–17	The Netherlands	[18,47]
	TeA	40	80–100	<5–1350	The Netherlands	[18,46,47]
	ALT	11	9	<14	Germany	[18]
	ATX-I	11	9	<45	Germany	[18]
	TEN	16	20–91	<3.7–800	Germany	[18,46]
Feed	BEA	1345	50–98	<2–2326	not specified	[4,38,39,40]
	ENN A	1315	0–87	<0.1–1745	not specified	[4,39,40]
	ENN A1	1315	12–95	<0.15–2216	not specified	[4,39,40]
	ENN B	1414	28–92	<0.3–1514	not specified	[4,39,40,50]
	ENN B1	1315	12–92	<0.2–1846	not specified	[4,39,40]
	MON	1315	3–79	<2–12,236	not specified	[4,39,40]
	AOH	264	0–80	17–221	not specified	[4,50,51]
	AME	264	1.5–82	<6–733	not specified	[4,50,51]
	TeA	83	65	not specified-1983	not specified	[4]

^a^ Including wheat, oats, barley and rye; ^b^ Sum of different ENNs or not specified; ^c^ Including tomato sauce, paste, pieces, concentrate, pieces and ketchup; ^d^ Including apple, apricot, carrot, citrus, currant, grape, grapefruit, multi fruit, orange, sour cherry and vitaminized (ACE) juice; ^e^ Including tea infusions, puree infant food and cereals.

**Table 2:** In vitro toxicity of emerging *Fusarium* and *Alternaria* mycotoxins.

Cell Line	Mycotoxin	Exposure Time	Exposure Dose (µM)	Effect	Reference
Caco-2 ^a^	BEA	0 min	1.5	ROS ^b^ generation	[5,6,56,66,67]
		24–72 h		IC_50_: 20.6–3.2 µM (MTT ^c^); IC_50_: 8.8–1.9 µM (NR ^d^)	
		24–72 h	1.5–3.0	LPO ^e^, ↓ GSH, ↑ GSSG, loss of mitochondrial membrane potential, cell cycle arrest in S and G2/M, apoptosis and necrosis	
		24 h	12	DNA damage	
	ENN A	<1 h	1.5–3.0	ROS generation	
		24–72 h		IC_50_: 9.3–0.46 µM	
		24–72 h	1.5–3.0	LPO, loss of mitochondrial membrane potential, cell cycle arrest in SubG0/G1 and (Sub)G2/M, DNA damage, apoptosis and necrosis	
	ENN A1	10 min	1.5	ROS generation	
		24–72 h		IC_50_: 12.3–0.46 µM	
		24–72 h	1.5–3.0	LPO, loss of mitochondrial membrane potential, DNA damage, cell cycle arrest in (Sub)G0/G1 and G2/M, apoptosis, necrosis	
	ENN B	10 min	3.0	ROS generation	
		48–72 h		IC_50_: 10.7–1.4 µM	
		24–72 h	1.5–3.0	LPO, loss of mitochondrial membrane potential, cell cycle arrest in (Sub)G0/G1, and G2/M, apoptosis, necrosis	
	ENN B1	5–10 min	1.5–3.0	ROS generation	
		48–72 h		IC_50_: 10.8–0.8 µM	
		24–74 h	1.5–3.0	LPO, loss of mitochondrial membrane potential, DNA damage, cell cycle arrest in (Sub) G0/G1, G2/M and S, apoptosis, necrosis	
	MON	72 h		IC_50_: 30.9 µg/mL	
	AOH	24 h	15–30	changes in MMP ^f^, ↓ G1 phase, ↑ S and G2/M phase, apoptosis, necrosis	
HT-29 ^g^	ENN A	24–48 h		IC_50_: 9.3–8.2 µM	[56]
	ENN A1	24–48 h		IC_50_: 9.1–1.4 µM	
	ENN B	24–48 h		IC_50_: ≥2.8 µM	
	ENN B1	24–48 h		IC_50_: 16.8–3.7 µM	
HCT116 ^g^	AOH			IC_50, 24h_: 65 µM ↓ early apoptotic and late apoptotic/necrotic cells, ROS generation PTP ^h^-dependent MMP caspase-cascade activation, activation of p53 protein expression	[68,69]
	AME			IC_50, 24h_: 120 µM apoptotic cell death, PTP-opening, induction of MMP, cytochrome c release caspase-cascade activation, ↑ p53 protein, ROS generation	
IPEC-J2 ^i^	BEA	24–72 h	5–10	TEER^j^ reduction (between −59% and −80%), no reduction of cell viability	[59]
	ENN A	72 h	5	TEER reduction (−70%), no reduction of cell viability	
	ENN A1	24–72 h	10	TEER reduction (between −29% and −74%), no reduction of cell viability	
	ENN B	48–72 h	2.5	TEER reduction (between −55% and −68%), no reduction of cell viability	
	ENN B1	48–72 h	5	TEER reduction (between −44% and −58%), no reduction of cell viability	
	ENN combinations		1.5	additive effect on TEER reduction	
	MON	72 h	5–10	no effect on TEER or viability	[59]
Hep-G2 ^k^	ENN A	24–48 h		IC_50_: 26.2–11.4 µM	[56,66]
	ENN A1	24–48 h		IC_50_: 11.6–2.6 µM	
	ENN B	24–48 h		IC_50_: >30 µM	
	ENN B1	24–48 h		IC_50_: 24.3–8.5 µM	
	MON	48–72 h		IC_50_: 39.5–24.1 µg/mL	
H295R ^l^	ENN B	72 h	10–100	↓ viability by 37%, ↑ S-phase, ↓ G0/G1phase, ↑ apoptosis ↓ HMGR, STAR, CYP11A, HSD3B2, CYP17A1 ↑ CYP1A1, MC2R, NR0B1, CYP21A2, CYP11B1, CYP19 ↓ progesterone, testosterone and cortisol; estradiol unaffected	[63,70,71]
	AOH		3.87	no influence on viability ↑ 7 proteins (FDX1, HSD3B, CYP21A2, SCAMP3, SOAT1, ARF6, RRP15) ↓ 15 proteins (ACTBL2, NUCKS1, EIF2B5, COX2, CRMP1, ABHD14A-ACY1, ATP5J, ACSF2, HN1, ETHE1, HIST1H1E, ACBD5, NPC1, NR5A1, TOMM7) upregulation mRNA for CYP21A2 and HSD3B ↑ G0/G1 and ↑ G2/M phase	
H29R ^l^	AOH			no effect on testosterone and cortisol levels ↑ progesterone and estradiol levels ↓ *NR0B1* gene ↑ *CYP1A1, MC2R, HSD3B2, CYP17, CYP21, CYP11B2, CYP19*	[72]
neonatal Leydig cells	ENN B		10–100	↓ viability by 20%, ↓ estradiol in unstimulated cells ↓ estradiol and testosterone in LH stimulated cells, probably due to cytotoxicity	[63]
human breast adenocarcinoma RGA cell line	AOH			agonistic estrogen response, relative estrogenic potential: 0.0004% and equivalent estrogenic quantity of 17β-estradiol: 2.9 fg/mL	[72]
cell free buffer	AOH			binding affinity to ERα: 10,000× lower compared to 17β-estradiol binding affinity to ERβ: 2500× lower compared to 17β-estradiol similar EC_50_	[73]
Ishikawa human endometrial adenocarcinoma cell line	AOH		2.5–10	↑ alkaline phosphatase mRNA and activity ↓ G1 phase and ↑ S and G2/M phase ↓ cell number due to inhibition of proliferation	[73]
porcine oocytes and embryos	BEA		>0.5	↓ rate of development of maturing oocyte and 2–4 cell stage embryo, activated oocytes and 2–4 cell stage embryos more sensitive than maturing oocytes, compromised cytoplasmic maturation and abnormal meiosis in oocytes, ↓ cumulus viability and progesterone synthesis, cumulus cells control intracellular BEA through MDR1 activity, in oocytes mitochondrial function was altered, altered gene expression in cumulus cells and oocytes, altered MDR1 activity in activated oocytes, ↓ viability embryo	[61]
pig granulosa cells	AOH		0.8–1.6	↓ cell viability, ↓ progesterone levels, ↓ P450scc ↓ α-tubulin, actin and EIF4a	[71]
	AME		0.8–1.6	↓ cell viability, ↓ progesterone levels, ↓ P450scc	
	TeA		6.4–100	no influence on viability no influence on progesterone concentrations	
bovine granulosa cells	BEA		3	↓ estradiol and progesterone production ↓ *CYP11A1* and *CYP19A1* mRNA	[62]
			6–10	↓ (fetal calf serum-induced) proliferation	
					
CHO-K1 ^m^	BEA	24–72 h		IC_50_: 10.7–2.2 µM combination of BEA + PAT ^n^, BEA + STG ^o^, BEA + PAT + STG: synergistic effect at low (IC < 1), additive effect at higher (IC 0.6–5.9) doses	[52,66,74]
	ENN A	24–72 h		>7.5–2.83 µM	
	ENN A1	24–72 h		8.8–1.65 µM	
	ENN B	24–72 h		11.0–2.44 µM	
	ENN B1	24–72 h		4.53–2.47 µM	
	ENN combinations	24 h		additive effects: A + B1, A1 + B, B + B1 synergistic effects: A + A1, A + B, A1 + B1, A1 + B1, A + A1 + B, A + A1 + B1, A1 + B + B1 (higher concentrations) antagonistic effects: A + A1 + B1, A1 + B +B1 (lower concentrations)	
	MON			IC_50_: >100 µg/mL	
					
					
THP-1 ^p^ monocyte	AOH	24–48 h	7.5–15	cell cycle arrest in S- and G2/M-phase	[55]
				↓ CD14 and CD11b upregulation during macrophage differentiation	
				↓ downregulation of CD71 during macrophage differentiation, ↓ TNF-α secretion due to ↓ gene expression	
				+DON: additive effect +ZEA: synergistic effect on macrophage differentiation	
CCRF-CEM ^q^	BEA	24 h	1	cytotoxicity, apoptosis	[54]
					
human lymphocytes	MON	48 h	10–25 15–25	chromosome breaks, chromatid breaks and exchanges, polyploidy, increase in sister chromatid exchanges and micronuclei frequency all effects were dose-dependent	[8]
human immature dendritic cells	BEA			IC_50_: 1.0 µM	[53]
	ENN B			IC_50_: 1.6 µM	
	MON		80	20% mortality, ↓ endocytosis, ↓ CD1a expression	
human mature dendritic cells	BEA			IC_50_: 2.9 µM, ↓ CCR7 expression, ↑ IL-10 concentration	[53]
	ENN B			IC_50_: 2.6 µM, ↓ CD80, CD86 and CCR7 expression, ↑ IL-10	
	MON		80	20% mortality	
human macrophages	BEA		≥0.5	IC_50_: 2.5 µM, ↓ endocytosis	[53,75]
	ENN B			IC_50_: 2.5 µM, ↓ endocytosis, ↑ CD71	
	MON			↓ endocytosis, ↓ CD71, ↓ HLA-DR	
	AOH	24 h	30	changed morphology: from round to elongated with dendrite-like protrusions ↑ CD83 and CD86 ↓ HLA-DR and CD68 ↑ secretion of TNFα and IL-6 ↓ endocytosis and ↓ autophagy double DNA strand breaks	
RAW 2654.7 mouse macrophage	AOH	24–48 h	30	changed morphology: from round to flattened, star-shaped or elongated spindle-shaped cells micronuclei, polyploidy, ↑ CD86, CD80, MHCII (T cell activation), ↑ CD11b ↑ mRNA of TNFα and IL-6, but only ↑ TNFα secretion, ↑ endocytosis	[75]
mouse hemidiaphragm preparation	BEA		5	inhibition (in) directly elicited tetanic muscle contraction; inhibition nerve-evoked and directly elicited muscle twitches, reduction amplitude and frequency of miniature endplate potentials	[60]
		1 h	7.5	inhibition directly elicited twitches, induction contracture, decrease resting membrane potential	
		1 h	10	complete block of (in) directly elicited isometric muscle contraction, amplitude reduction of directly elicited muscle twitch, decrease resting membrane potential	
C5-O ^r^	MON	72 h		IC_50_: 34.2 µg/mL	[66]
V79 ^s^	MON	72 h		IC_50_: >100 µg/mL	[62,66,73]
	AOH		5–50	induction of micronuclei cell cycle arrest in G2 and S phase	

↓ decrease; ↑ increase; ^a^ human adenocarcinoma colon cells; ^b^ reactive oxygen species; ^c^ tetrazolium salt reduction assay; ^d^ Neutral Red assay; ^e^ lipid peroxidation; ^f^ mitochondrial membrane permeabilization; ^g^ human colon carcinoma cells; ^h^ permeability transition pore; ^i^ intestinal porcine epithelial cells from the jejunum; ^j^ transepithelial electrical resistance; ^k^ human hepatocellular carcinoma cells; ^l^ human adrenocortical carcinoma cells; ^m^ Chinese hamster ovary cells; ^n^ patulin; ^o^ sterigmatocystin; ^p^ human acute monocyte leukemia cell line; ^q^ human leukemia cells; ^r^ Balb/c mice keratinocyte cells; ^s^ Chinese hamster lung fibroblast.

**Table 3:** In vivo toxicity of moniliformin and *Alternaria* mycotoxins.

Animal Species	Mycotoxin	Route of Exposure	Exposure Time	Exposure Dose	Effect	Reference
mouse	MON	po-ip	1x		LD_50_: 20.9 (♀) 29.1 (♂) mg/kg bw (ip), survivors clinically healthy	[13,92]
	TeA	iv-po	1x	0–398 mg/kg bw	LD_50_: 76–162 (iv) and 81–209 (po), vomiting, diarrhea, hemorrhages, death	
Sprague-Dawley rats	MON	po	1x	5 mg/kg bw	no clinical signs	[13,98,99]
			1x	10 mg/kg bw	↓ activity for 24 h, respiratory changes, trembling, piloerection, complete recovery within 48 h	
			1x	25–50 mg/kg bw	respiratory and cardiovascular changes, collapse, convulsion and death within 48–83 min	
			28 days	3–6 mg/kg bw	no clinical symptoms, no effect on leucocyte and red blood cell counts, food and water consumption or organ and body weights, ↓ phagocytic activity of neutrophils	
	TeA	iv-po	1x	0–398 mg/kg bw	LD_50_: 83–157 (iv) and LD_50_: 168–240 (po), vomiting, diarrhea, hemorrhages, death	
Syrian golden hamster	AME	ip	1x	200 mg/kg bw	severe necrosis and coalescence of visceral organs lethargy, breathing difficulties, flaccid hind limbs↑ resorptions and ↓ fetal weight	[100]
chicken embryo	MON	injection	1x		LD_50_: 2.8 µg/egg, no gross teratogenic effects in survivors	[92]
	TeA	injection	1x	150–1500 µg/egg	dose-related mortality, LD_50_: 548 µg/egg	[101]
1-day old chicken	MON	po	1x	0–16 mg/kg bw	LD_50_: 5.4 mg/kg bw (crop intubation), survivors clinically healthy	[92]
broiler chickens	MON	feed	21 days	200 mg/kg feed	death (56%) ↑ kidney, heart and liver weight ↑ serum albumin, total protein and aspartate aminotransferase	[11,14,102]
				100 mg/kg feed	↓ feed intake and body weight gain ↑ hearth weight ↑ kidney weight if feed also contained 200 mg FB_1_/kg ↑ incidence of large pleomorphic cardiomyocyte nuclei loss of cardiomyocyte cross striations mild focal renal tubular mineralization	
		feed	42 days (day 7–49)	50 mg/kg feed	mortality (13.3%) ↑ feed consumption, ↓ body weight gain, ↓ feed conversion ↑ relative heart and proventriculus weight, ↓ mean corpuscular volume ↑ serum gamma glutamyltransferase activity loss of cardiomyocyte cross striations ↑ cardiomyocyte nuclear size	
				25 mg/kg feed	mortality (7.8%) ↑ serum gamma glutamyltransferase activity	
	TeA	po	21 days	1.25–2.5 mg/kg bw	↓ weight gain and feed efficiency hemorrhages, erosions of the gizzard, pale mottled spleens, edema of the myocardium, microscopic congestions of blood vessels and hemorrhages	
White leghorn chicken	TeA	po	1x		LD_50_: 37.5 mg/kg bw with hemorrhages of the musculature of the thigh, breast, heart and subcutaneous tissues	[14]
			21 days	0.63 mg/kg bw	pathological changes in spleen and gizzard but no extensive hemorrhages microscopic congestions of blood vessels and hemorrhages	
			21 days	1.25–2.5 mg/kg bw	↓ weight gain and feed efficiency hemorrhages, erosions of the gizzard, pale mottled spleens, edema of the myocardium, microscopic congestions of blood vessels and hemorrhages	
turkeys	MON	feed	91 days (day 7–98)	25, 37.5, 50 mg/kg feed	↑ relative heart weight	[102]
				37.5, 50 mg/kg feed	↑ relative liver weight	
				50 mg/kg feed	loss of cardiomyocyte cross striations ↑ cardiomyocyte nuclear size, ↑ number of cardiomyocyte mitotic figures	
turkey poults	MON	feed	21–28 days	100 mg/kg feed	↓ feed intake, body weight gain, feed efficiency, ↓ relative thymus, bursa and spleen weights, ↓ primary and secondary antibody response to inactivated Newcastle disease virus, ↓ systemic clearance of *E. coli*	[103]
Japanese quail	MON	feed	35 days	100 mg/kg feed	cardiomegaly, myocardial congestion, hypertrophy, myocardial disarray, ↑ mitochondria, resulting in separation of muscle fibers, swollen and deformed mitochondria with degenerative changes. Congestion, hemorrhages and degenerative changes more pronounced and extensive disruption of muscle fibers and destruction of Z-bands when feed contained both MON and fumonisin B1. Death.	[12]
barrow	MON	feed	28 days	100 mg/kg feed	acute mortality due to apparent cardiac failure ↓ body weight gain ↓ body weight gain, feed consumption and feed efficiency when feed also contained FB_1_	[104]
dog	TeA	iv-po	1x	25–50 mg/kg bw	severe tachycardia, massive diffuse hemorrhages, (bloody) diarrhea	[13]
		iv	3x	20 mg/kg bw	severe hemorrhagic gastro-enteropathy, death	
		iv-po	6–30 days	0.0625–11.2 mg/kg bw	salivation, emesis, tachycardia, hemorrhagic gastro-enteropathy, death	
monkey	TeA	iv	3x	20 mg/kg bw	severe hemorrhagic gastro-enteropathy, death	[13]
		iv-po	7–45 days	11.2–89.6 mg/kg	salivation, emesis, hemorrhagic gastro-enteropathy, death	

↓ decrease; ↑ increase.
